# Randomized controlled trials in pediatric critical care: a scoping review

**DOI:** 10.1186/cc13083

**Published:** 2013-10-29

**Authors:** Mark Duffett, Karen Choong, Lisa Hartling, Kusum Menon, Lehana Thabane, Deborah J Cook

**Affiliations:** 1 Departments of Pediatrics and Clinical Epidemiology and Biostatistics, McMaster University, 1280 Main Street West, Hamilton, ON L8S 4L8, Canada; 2 Department of Pediatrics and Alberta Research Centre for Health Evidence, University of Alberta, 116 Street and 85 Avenue, Edmonton, AB T6G 2R3, Canada; 3 Departments of Pediatrics and Epidemiology and Community Medicine, University of Ottawa, 75 Laurier Avenue East, Ottawa, ON K1N 6N5, Canada; 4 Departments of Clinical Epidemiology and Biostatistics, and Pediatrics, Biostatistics Unit, St Joseph’s Healthcare-Hamilton, McMaster University, Hamilton1280 Main Street West, ON L8S 4L8, Canada; 5 Departments of Medicine and Clinical Epidemiology and Biostatistics, 462 McMaster University, 1280 Main Street West, Hamilton, ON L8S 4L8, Canada

## Abstract

**Introduction:**

Evidence from randomized controlled trials (RCTs) is required to guide treatment of critically ill children, but the number of RCTs available is limited and the publications are often difficult to find. The objectives of this review were to systematically identify RCTs in pediatric critical care and describe their methods and reporting.

**Methods:**

We searched MEDLINE, EMBASE, LILACS and CENTRAL (from inception to April 16, 2013) and reference lists of included RCTs and relevant systematic reviews. We included published RCTs administering any intervention to children in a pediatric ICU. We excluded trials conducted in neonatal ICUs, those enrolling exclusively preterm infants, and individual patient crossover trials. Pairs of reviewers independently screened studies for eligibility, assessed risk of bias, and abstracted data. Discrepancies were resolved by consensus.

**Results:**

We included 248 RCTs: 45 (18%) were multicentered and 14 (6%) were multinational. Trials most frequently enrolled both medical and surgical patients (43%) but postoperative cardiac surgery was the single largest population studied (19%). The most frequently evaluated types of intervention were medications (63%), devices (11%) and nutrition (8%). Laboratory or physiological measurements were the most frequent type of primary outcomes (18%). Half of these trials (50%) reported blinding. Of the 107 (43%) trials that reported an *a priori* sample size, 34 (32%) were stopped early. The median number of children randomized per trial was 49 and ranged from 6 to 4,947. The frequency of RCT publications increased at a mean rate of 0.7 RCTs per year (*P*<0.001) from 1 to 20 trials per year.

**Conclusions:**

This scoping review identified the available RCTs in pediatric critical care and made them accessible to clinicians and researchers (http://epicc.mcmaster.ca). Most focused on medications and intermediate or surrogate outcomes, were single-centered and were conducted in North America and Western Europe. The results of this review underscore the need for trials with rigorous methodology, appropriate outcome measures, and improved quality of reporting to ensure that high quality evidence exists to support clinical decision-making in this vulnerable population.

## Introduction

Evidence from randomized controlled trials (RCTs) is required to guide treatment of critically ill children. There are fewer RCTs in pediatrics when compared to adult medicine; in reviews of RCTs published in general and specialist medical journals only 14% of trials enrolled exclusively children [1,2]. Moreover, while the methodological quality of pediatric RCTs appears to be improving, 37 to 59% were still at high risk of bias [3-5]. Finally, the focus of published pediatric RCTs may not align with the frequency or importance of the conditions seen in clinical practice. For example, in pediatric primary care there is discordance between the conditions studied and the frequency seen in clinical practice: 23% of Cochrane systematic reviews relevant to pediatrics focused on asthma, which represents 3 to 5% of children’s primary care visits [6].

The extent of these challenges in pediatric critical care has not previously been examined. An example from critical care is the Surviving Sepsis Campaign’s International Guidelines for Management of Severe Sepsis and Septic Shock [7]. These guidelines highlight the limited quality of evidence available in pediatric critical care to support clinical decision-making. The consensus committee was able to make 76 recommendations for adults, but only 22 pediatric-specific recommendations. Not only are there fewer pediatric recommendations, but the quality of the evidence informing these pediatric recommendations is lower; 3 (14%) of the pediatric recommendations were supported by high/moderate evidence as compared to 41 (54%) of those for adults.

To effectively apply the results of pediatric critical care RCTs, it is imperative that clinicians can easily and efficiently find these publications. However, clinicians are not typically trained to conduct the complex literature searches required to find pediatric RCTs; even a highly specific search strategy yielded only 56% of citations relevant to children [8]. Challenges in locating relevant pediatric RCTs are likely to increase as the number of adult RCTs increases faster than the number of pediatric RCTs in both general medical journals (4.7 RCTs per year vs. 0.4 RCTs per year) and in specialist journals (91 RCTs per year vs. 17 RCTs per year) [1,2]. There also are few tools, resources or reviews to help clinicians quickly access or identify the available RCTs in pediatric critical care.

A scoping review systematically maps a broad and diverse body of research evidence [9]. We conducted this scoping review to systematically identify and describe RCTs in pediatric critical care and make them readily accessible to clinicians and researchers.

## Materials and methods

### Trial eligibility

We included RCTs and quasi-randomized trials that reported the effect of any intervention on children or their families in a pediatric intensive care unit. We used the authors’ definitions of pediatric and only included trials in which critically ill children were a subgroup if the demographic and outcome data for the critically ill children were reported separately. We considered a unit to be an intensive or critical care unit if the authors described it as such and if it had the capacity to provide mechanical ventilation. We included trials in all languages. We excluded trials enrolling exclusively preterm infants or infants in a neonatal intensive care unit, individual patient crossover trials and those only published as abstracts. For trials reported in multiple publications we used the most recent publication. We excluded substudies and secondary publications of included RCTs.

### Searching

We searched MEDLINE, EMBASE, LILACS and CENTRAL from inception to April 16, 2013. To identify RCTs we used previously tested search strategies for MEDLINE (the Cochrane Highly Sensitive Search Strategy, sensitivity- and precision-maximizing version [10]) EMBASE [11] and LILACS [12]. To identify studies enrolling children in MEDLINE we used a previously tested strategy [8] and adapted for the other databases. We then added search terms related to pediatric critical care. Additional file 1: Appendix A contains the full search strategies (see Additional file 1). To identify other potentially relevant trials, we also examined the reference lists of all included RCTs, systematic reviews identified by our searches, and the researchers’ personal files.

### Study selection and data extraction

We developed an electronic data collection tool using DistillerSR™ (Evidence Partners Incorporated, Ottawa, ON, Canada) and an accompanying screening and data extraction manual. To increase consistency among reviewers, all reviewers screened the same 50 publications, discussed the results and amended the screening and data extraction manual before beginning screening for this review. Nine reviewers working in pairs sequentially evaluated the titles, abstracts and then full text of all publications identified by our searches for potentially relevant publications. Reviewers then worked in pairs to independently and in duplicate extract data from the included trials using a pretested electronic data collection tool. We recruited other individuals with a clinical or research methodology background to screen and extract data from non-English trials. We were not able to complete duplicate data extraction for two trials because of the language of publication. We resolved disagreements on study selection and data extraction by consensus and discussion with other reviewers if needed. We extracted data from the main trial publication and also any referenced published protocols and supplemental materials.

### Risk of bias assessment

We used the Cochrane Risk of Bias Tool to describe the risk of bias for the included trials [13]. This tool rates each trial as low, unclear, or high risk of bias for each of the following factors: sequence generation, allocation concealment, blinding of participants and personnel, blinding of outcome assessment, incomplete outcome data, selective outcome reporting, and other sources of bias. We then classified the overall risk of bias for each trial as low (low risk of bias in all domains), high (high risk of bias in at least one domain), or unclear.

### Statistical analysis

We used the kappa statistic to assess agreement between reviewers and considered values of 0.6 or greater to indicate substantial agreement [14]. In summarizing the characteristics of included systematic reviews and RCTs we reported continuous data as medians (interquartile range (IQR), and binary data as count (percent). We used linear regression to evaluate the changes over time in the number of trials published. We used the Mann-Whitney *U* test to compare the number of children randomized in trials that reported early stopping and those that did not and those that reported funding and those that did not, hypothesizing that sample sizes would not be statistically different. We used Fisher’s exact test to compare the proportion of trials that were stopped early among those reporting funding with those that did not, hypothesizing that funded trials would be less frequently stopped early. We used alpha = 0.05 as the criterion for statistical significance. We used SPSS Statistics Version 21 (IBM Corp. Armonk, NY, USA) to perform the statistical analysis.

## Results

### Trial publication

**Figure 1:**
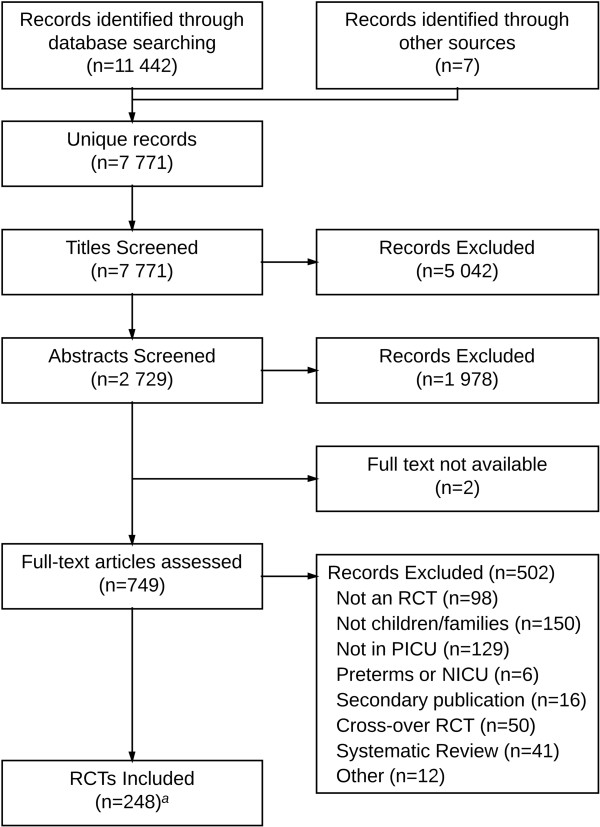
**Review flow diagram.**^*a *^One publication included two related RCTs: a single-center and a multicenter trial with different inclusion and exclusion criteria. RCTs, randomized controlled trials; SR, systematic review; PICU, pediatric intensive care unit; NICU, neonatal intensive care unit.

**Figure 2:**
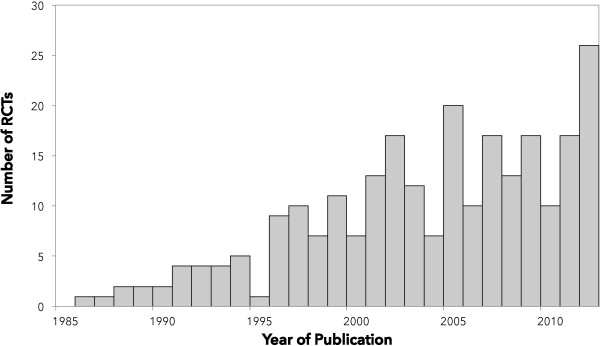
**Number of pediatric critical care randomized controlled trials published per year (1986 to 2012).** An additional 12 trials were published between January and April 2013.

We included 248 RCTs randomizing a total of 27,013 children out of 7,771 unique publications screened. Figure 1 shows the flow of studies through the review process and Additional file 2: Appendix B lists all of the included trials (see Additional file 2). Chance-corrected agreement for study inclusion was almost perfect (kappa = 0.93, 95% CI 0.91 to 0.96). The included RCTs were published in 89 different journals. The five journals that published the highest number of trials: *Pediatric Critical Care Medicine* (31), *Critical Care Medicine* (29), *Intensive Care Medicine* (19), *Journal of Pediatrics* (7) and *The New England Journal of Medicine* (7) published 32% of the included trials. Almost half (46%) were published in the 17 journals with a specific critical care focus. Sixteen trials (6%) were published in six languages other than English. Figure 2 shows the number of trials published per year. The number of trials published per year increased from one in 1986 to twenty in 2012. The mean rate of increase was 0.7 RCTs per year (95% CI = 0.5 to 0.8; *P* <0.001; r^2^ = 0.76).

### Description of included trials

**Figure 3:**
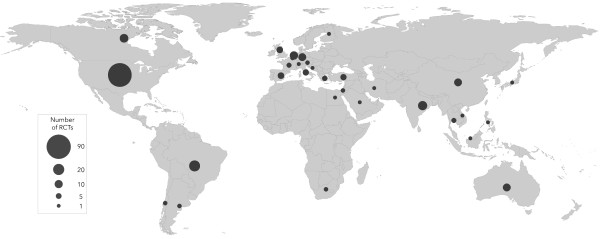
**Number of published pediatric critical care randomized controlled trials per country (1986 to 2013).** This map shows the country where each trial was conducted. We used the country of the primary author for multinational trials.

Table 1 shows the characteristics of included trials. The majority (82%), were single-centered and the median (IQR) number of centers participating in the multicentered trials was five (two to eight) and varied from two to one hundred and four. Trials were conducted in 31 different countries (Figure 3). The majority, 170 (69%), were from North America (104 trials) or Western Europe (66 trials) and were concentrated in a small number of countries. With respect to the number of trials conducted, the top five countries (Brazil, Canada, India, The Netherlands and the United States) conducted 59% of the RCTs. When measured by the number of children randomized, the top five countries (Australia, Brazil, Canada, Germany, and the United States) enrolled 69% of the children randomized. The countries with the highest median number of children randomized per trial were Argentina (149), Spain (122), Israel (107) and The Netherlands (74).

**Table 1:** Methodological characteristics of 248 pediatric critical care trials (1986 to 2013)

**Characteristics**	**RCTs n (%)**
Multicentered	45 (18)
Multinational	14 (6)
Number of children randomized	49 (30 to 93)^*a*^
	Min 6, Max 1199^*b*^
Number of children included in analysis	44 (28 to 80)^*a*^
	Min 6, Max 980^*b*^
Type of primary outcome	
Laboratory or physiological	44 (18)
Clinical complications	29 (12)
Duration of ventilation	14 (6)
Severity of illness score	14 (6)
Clinical success	13 (5)
Process of care	13 (5)
Mortality	5 (2)
Other	32 (13)
Not reported	82 (33)
Source of funding	
Noncommercial only	102 (41)
Commercial only	28 (11)
Both commercial and noncommercial	12 (5)
None	5 (2)
Not reported	101 (41)
Non-financial commercial support only	21 (8)
Early stopping	
No	79 (32)
Yes	34 (15)
Futility, recruitment or funding	23 (9)
Benefit	5 (2)
Harm	2 (1)
Unclear	4 (2)
Unclear	133 (54)

^*a*^Median (IQR, interquartile range); ^*b*^this maximum does not include the one cluster RCT that randomized ten PICUs at five sites and included 4,947 children [15].

All trials randomized individual children except one cluster RCT which randomized ten PICUs in five centers [15]. Approximately half (54%) of the 183 trials that reported the duration of enrolment were completed in less than two years. Thirty-eight trials (15%) reported in the publication that the trial had been registered. A significant proportion of trials (41%) did not report their funding source and 16% had at least some industry funding.

Table 2 shows the populations studied. Families were the target of the intervention in two trials: the remainder focused on individual children. The mean age of participants in the 150 of 248 trials (61%) in which the mean age was reported was 4.2 years (minimum 1 month, maximum 14 years). Medical and surgical patients were similarly represented (23% and 24% respectively) with cardiac surgery patients representing the commonest group of patients studied (19%). Table 3 shows the types of conditions studied. Respiratory conditions were the most commonly studied (24%), followed by cardiac (16%) and gastrointestinal (15%) conditions. The majority of trials evaluated medications (Table 4) with analgesics and sedatives being the most frequent group of drugs evaluated (8%).

**Table 2:** Categories of patients enrolled in 248 pediatric critical care trials (1986 to 2013)

**Patient type**	**RCTs n (%)**
Medical/Surgical	106 (43)
Medical only	56 (23)
Bronchiolitis	16 (6)
Sepsis/Shock	13 (5)
Asthma	8 (3)
Other medical	19 (8)
Surgical only	60 (24)
Cardiac surgery	46 (19)
Noncardiac surgery	14 (6)
Trauma/Burns only	26 (10)
Traumatic brain Injury	13 (5)
Burns	13 (5)

**Table 3:** Types of conditions studied in 248 pediatric critical care trials (1986 to 2013)

**Indication**	**RCTs n (%)**
Respiratory	59 (24)
ARDS/ALI	14 (6)
Post extubation stridor	11 (4)
Ventilation and weaning	11 (4)
Bronchiolitis	9 (4)
Asthma	8 (3)
Other	6 (2)
Cardiac	19 (8)
Pulmonary hypertension	7 (3)
Other	12 (5)
Gastrointestinal	35 (14)
Nutrition	20 (8)
Stress ulcer prophylaxis	8 (3)
Feeding tube placement	5 (2)
Other	2 (1)
Infection	33 (9)
Central nervous system	26 (10)
Sedation/analgesia	22 (9)
Seizures	4 (2)
Sepsis/shock	20 (8)
Trauma	13 (5)
Traumatic brain injury	10 (4)
Burns	3 (1)
Fluids	12 (5)
Hematologic	9 (4)
Anemia	5 (2)
Thrombosis	4 (2)
Hyperglycemia	6 (2)
Other	16 (6)

ARD, acute respiratory distress syndrome; ALI, acute lung injury.

**Table 4:** Types of interventions studied in 248 pediatric critical care trials (1986 to 2013)

**Type of intervention**	**RCTs n (%)**
Medication	155 (63)
Anti-infectives	20 (8)
Analgesics/sedatives	21 (8)
Vasoactives	19 (8)
Corticosteroids	12 (5)
Medical gases	11 (4)
Surfactants	8 (3)
Acid suppression	8 (8)
Bronchodilators	7 (3)
Insulin	6 (2)
Anticoagulants	3 (1)
Anticonvulsants	3 (1)
Diuretics	3 (1)
Other	34 (14)
Devices	27 (11)
IV catheters, care or placement	7 (3)
Feeding tube placement	6 (2)
Ventilator or other respiratory	5 (2)
IV pumps and infusions	3 (1)
Other	6 (2)
Nutrition	21 (8)
Ventilation	17 (7)
IV fluids	14 (6)
Blood products	13 (5)
Hypothermia	6 (2)
Physiotherapy	5 (2)
Psychosocial	2 (1)
Other	6 (2)
More than one type of intervention	21 (9)

We could determine the primary outcome in 166 (67%) trials (Table 1). Of these, laboratory and physiological primary outcomes were the most frequently reported. Mortality was the primary outcome measure in only 2% of trials. Of the 148 trials (60%) reporting mortality, the median (IQR) mortality was 8% (1 to 15%), varying from 0% to 94%. The mortality was 0% in 33 trials (13%). We could assess the statistical significance of the primary outcome (using the authors definitions or *P* <0.05 if not defined) in 133 RCTs that compared two interventions. In 67 (50%) of trials, the results were statistically significant: 62 (93%) of these favored the experimental intervention.

### Sample size

**Figure 4:**
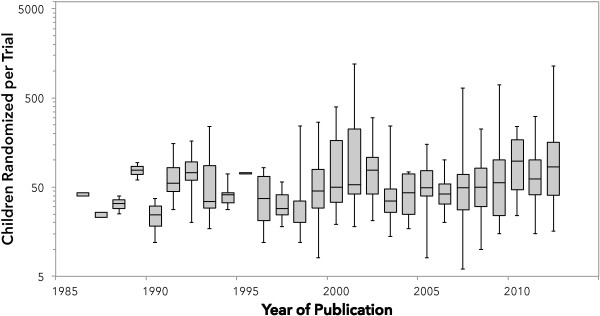
**Number of children randomized in 236 pediatric critical care randomized controlled trials (1986 to 2012).** The center of each box indicates the median number of children randomized in the RCTs published in each year. The bottom and top of each box indicate the 1^st^ and 3^rd^ quartile respectively and the whiskers indicate the maximum and minimum values for RCTs in that year. An additional 12 trials published between January and April 2013.

The number of children randomized in individual-patient RCTs varied from 6 to 1,199; the one cluster RCT randomized 10 PICUs and included 4,937 children. There were six trials randomizing more than 500 (including three randomizing more than 1,000) children, all published since 2001. The median (IQR) number of children randomized per trial was 49 (30 to 93). The median sample sizes varied from 23 to 98 in the period 1986 to 2013 and was less than 50 for 16 of these 28 years of publication (Figure 4). The number of children randomized per year increased from 40 in 1986 to 3,806 in 2012. The mean rate of increase was 82 children per year (95% CI = 49 to 115; *P* <0.001; r^2^ = 0.49). A total of 134 trials (54%) included all randomized children in the analysis. The median (IQR) number of children randomized in the 142 RCTs that reported a funding source compared to trials that did not was not significantly different: 44 (80 to 32) compared to 52 (30 to 100), *P* = 0.24. We also evaluated the completeness of follow-up and early stopping among these RCTs. The mean proportion of randomized children who were not included in the analysis was 6% and varied from 0 to 59%. Thirty-four trials (15% of all 248 trials) were stopped early, most frequently for futility or recruitment problems (Table 1). Of the 107 trials that reported a planned sample size, 32% were stopped early. A total of 17% of the 142 trials reporting any funding were stopped early, compared to 8% of those who did not report funding (*P* = 0.04). The median (IQR) number of children randomized in trials that were stopped early was 50 (26 to 134) and 49 (30 to 84) in those that were not reported to be stopped early (*P* = 0.71).

### Risk of bias

**Figure 5:**
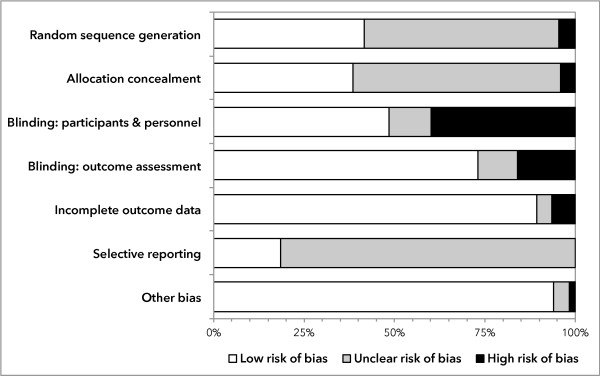
**Risk of bias in 248 pediatric critical care randomized controlled trials (1986 to 2013).** We used the Cochrane Risk of Bias Tool to describe the risk of bias for the included trials.

Figure 5 presents the risk of bias assessments for the individual domains of the Cochrane Risk of Bias tool. The overall risk of bias was low for 11 (4%) trials, high for 108 (44%) and unclear for the remaining 129 (44%). All trials at low risk of bias were published since 2006. Blinding was only reported in half the trials (125/248). Nine (6%) trials were quasi-randomized trials: using a process such as alternation or date of admission to assign participants to treatment groups.

## Discussion

Scoping reviews can be used ‘to examine the extent, range, and nature of research activity; to determine the value for undertaking a full systematic review; to summarize and disseminate research findings; and to identify research gaps in the existing literature’ [16]. In this scoping review we found 248 pediatric critical care RCTs, from 31 countries, published in 7 languages over 28 years. The majority of these RCTs were single-centered, focused on intermediate or surrogate outcomes and were small in sample size. Important aspects of their methodology and reporting remain less than optimal. As part of this review we have created a publicly accessible online database of these trials including key methodological features and links to the original reports [17].

There are gaps in the body of pediatric critical care RCT research. For example, rehabilitation and the needs of parents coping with their child’s illness are relevant for almost all critically ill children, yet there are only two trials focusing on each of these. Similarly, pharmacological interventions were studied in the majority of RCTs; although 27% of RCTs studied devices, there were no trials focused on renal replacement therapy or extracorporal membrane oxygenation. Pediatric critical care clinicians, researchers and decision-makers can use the results of this review as part of a process to evaluate unmet needs and set research priorities, both in terms of the focus and design of additional RCTs and other research designs. There are also important limitations to the reporting of these trials; for example, only two-thirds reported the primary outcome and less than half reported the planned sample size or the funding source.

When compared to the findings of a random sample of 300 pediatric RCTs published in 2007, pediatric critical care RCTs were less frequently multicentered (18% vs. 35%), randomized fewer children (median 49 vs. 83), and had a lower proportion assessed as having a low risk of bias (4% vs. 8%) [5]. Pediatric critical care RCTs are also smaller and less common than adult critical care RCTs. A systematic review of adult critical care RCTs with clinical or economic primary outcomes published in 16 prominent journals included 127 RCTs published between 2007 and 2012 [18]. We identified 79 pediatric RCTs over the same time period. Compared to these adult RCTs, pediatric RCTs randomized fewer participants (mean 109 vs. 519), and were less frequently multicentered (18% vs. 60%). Another systematic review of RCTs evaluating nutritional interventions in critically ill adults included 207 RCTs randomizing a mean of 112 adults in the period 1980 to 2008 [19]. Using similar criteria, we found 17 trials randomizing a mean of 48 children in the same time period.

We found that pediatric RCTs are generally small, single-centered, and primarily measured short-term laboratory and physiological outcomes. This raises two important issues for clinicians, researchers and funding agencies to consider. The first question is whether or not the small sample size matters: were these studies able to definitively answer the question posed? This is unclear for many trials included in this review as 57% did not report the planned sample size. The second question is did these trials use appropriate outcome measures? Trials focusing on surrogate or intermediate outcomes can be used to inform the design and conduct of future studies using patient-important outcomes or when a trial with patient-important outcomes is not feasible [20]. Further research should focus on assessing if these RCTs lead to subsequent trials focusing on patient-important outcomes or if the outcomes used are indeed appropriate surrogates for more patient-important outcomes [21]. If many trials are indeed too small to generate clear results, further research needs to be done to identify the barriers to conducting larger trials and methods to overcome this limitation. A previous mixed-methods study of pediatric trialists identified a lack of research training, negative research culture and logistical challenges as barriers to conducting methodologically rigorous pediatric RCTs [22]. One important factor we identified is certainly feasibility, as 27 trials in this review (11% of all trials, 22% of those reporting an *a priori* sample size) were stopped early for reasons such as enrollment challenges or futility.

Strengths include the comprehensive search strategy to identify relevant trials and incorporation of trials published in any language. For each study we assessed the clinical and methodological features, and the completeness and transparency of reporting. We have also made publicly available data from the included trials and links to the full-text publications [17]. This is updated quarterly. The public availability of the results of this scoping review increases the ability of clinicians and pediatric critical care researchers to easily access pre-appraised, relevant RCTs. Finally, by synthesizing the methodological features of, and identifying gaps in, the body of pediatric critical care research, it will allow researchers and funding agencies to prioritize trial designs to fill the gaps we identify in the conditions studied, trial methods, interventions, and the outcomes assessed.

This scoping review has some limitations. The relevance of this review to clinicians in some resource-limited areas may be limited, as *a priori*, we excluded trials that were conducted in settings where mechanical ventilation was not available. We limited this review to RCTs conducted in a pediatric ICU and acknowledge that that some trials conducted in other populations such as critically ill adults or neonates, or in other settings such as prehospital, the emergency department, or the operating room may also inform the care of children in the ICU. Our objective in this review was to identify and describe pediatric critical care RCTs. Other research designs are also relevant to pediatric critical care practice, but are beyond the scope of a single review to include all the potentially relevant research. To focus on trials most likely to inform clinical practice and to improve the feasibility of this review we also excluded individual patient crossover trials.

## Conclusions

This pediatric critical care scoping review identified the available RCTs and made them accessible to clinicians and researchers. Most RCTs focused on medications and intermediate or surrogate outcomes, were single-centered and were conducted in North America and Western Europe. While the number of published trials is increasing over time, the sample size is not. The results of this review underscore the need for trials with rigorous methodology, appropriate outcome measures, and improved quality of reporting in pediatric critical care. Such trials on a broad range of topics relevant to pediatric critical illness are required to ensure that more rigorous evidence exists to support clinical decision-making in this vulnerable population.

## Key messages

• This scoping review identified the available RCTs in pediatric critical care and made them accessible to clinicians and researchers (http://epicc.mcmaster.ca).

• Most RCTs focused on medications and intermediate or surrogate outcomes, were single-centered and were conducted in North America and Western Europe.

• While the number of published trials is increasing over time, the sample size is not.

• The results of this review underscore the need for trials with rigorous methodology, appropriate outcome measures, and improved quality of reporting.

## Abbreviations

ALI: Acute lung injury; ARDS: Acute respiratory distress syndrome; CI: Confidence interval; IQR: Interquartile range; NICU: Neonatal intensive care unit; PICU: Pediatric intensive care unit; RCT: Randomized controlled trial; SR: Systematic review.

## Competing interests

The authors have no competing financial or nonfinancial interests with this study.

## Authors’ contributions

MD conceived of and coordinated this study, participated in its design and data collection, performed the analysis, and drafted the manuscript. KC participated in the design, data collection and helped to draft the manuscript. LH participated in the design, data collection and helped to draft the manuscript. KM participated in the design, data collection and helped to draft the manuscript. LT participated in the design, provided statistical expertise and helped to draft the manuscript. DC participated in the design, data collection and helped to draft the manuscript. All authors read and approved the final manuscript.

## Supplementary Material

Additional file 1 **Appendix A.** Search strategies. Click here for file

Additional file 2 **Appendix B.** Included trials. Click here for file
