# Supplementary material for: Randomized controlled trials in pediatric critical care: a scoping review
Source: Crit Care. 2013 Oct 29;17(5):R256. doi: 10.1186/cc13083 (PMC4057256; doi:10.1186/cc13083)
Supplement: Additional file 1 — Appendix A. Search strategies. [file cc13083-S1.pdf]

Appendix A: Search Strategies

MEDLINE

1. intensive care units, pediatric/
2. ((critical$ or intensive) adj2 (care or ill$)).mp.
3. (picu or icu or pccu).mp.
4. or/1-3
5. child:.mp.
6. adolescent:.mp.
7. infan:.mp.
8. or/5-7
9. (neonat$ or newborn or NICU or "low birth$" or VLBW or LBW or birthweight or preterm or "pre-term" or prematur$).ti.
10. randomized controlled trial.pt.
11. controlled clinical trial.pt.
12. randomized.ab.
13. placebo.ab.
14. clinical trials as topic.sh.
15. randomly.ab.
16. trial.ti.
17. or/10-16
18. exp animals/ not humans.sh.
19. (4 and 8 and 17) not (9 or 18)

EMBASE

1. exp intensive care unit/
2. exp intensive care/
3. ((critical$ or intensive) adj2 (care or ill$)).mp.
4. (picu or icu or pccu).mp.
5. or/1-4
6. child:.mp.
7. adolescent:.mp.
8. infan:.mp.
9. or/6-8
10. newborn intensive care/ or (neonat$ or newborn or NICU or "low birth$" or VLBW or LBW or birthweight or preterm or "pre-term" or prematur$).ti.
11. exp animals/ not humans.sh.
12. random:.tw.
13. placebo:.mp.
14. double-blind:.tw.
15. or/12-14
16. (5 and 9 and 15) not (10 or 11)

CENTRAL

#1 picu or icu or pccu
#2 MeSH descriptor Intensive Care Units, Pediatric, this term only
#3 (critical* or intensive) near/2 (care or ill or illness)
#4 child* or infan* or adolescent*
#5 pediatric* or paediatric*
#6 (neonat* or newborn or nicu or preterm or "pre-term" or prematur* or "low birth*" or LBW or VLBW or birthweight):ti
#7 ((#1 OR #2 OR #3) AND (#4 OR #5))
#8 (#7 AND NOT #6)

LILACS

(MH:"Intensive Care Units, Pediatric" OR MH:"Intensive Care " OR MH: "Critical Care" OR "Cuidados Críticos" OR "Cuidados Intensivos" OR "Intensive care" OR "Critical care" OR "critically ill" OR "critical illness" OR PICU OR "UTI pediatrica" OR "Unidade de Terapia Intensiva") AND (MH:"Infant" OR MH:"Child, Preschool" OR MH:"Child" OR Preescolar$ OR Pré-Escolar$ OR Niño$ OR Criança$ OR Infant$ OR Lactante$ OR child$ OR pediatric$ OR paediatric$)
