# Supplementary material for: Randomized controlled trials in pediatric critical care: a scoping review
Source: Crit Care. 2013 Oct 29;17(5):R256. doi: 10.1186/cc13083 (PMC4057256; doi:10.1186/cc13083)
Supplement: Additional file 2 — Appendix B. Included trials. [file cc13083-S2.pdf]

## Appendix B: Included Trials


1. Aanpreung P, Vanprapar N, Susiva C, et al.: A randomized clinical trial comparing the efficacy of ranitidine and famotidine on intragastric acidity in critically ill pediatric patients. *J Med Assoc Thai* 1998; 81:185–189

2. Adelson PD, Ragheb J, Muizelaar JP, et al.: Phase II clinical trial of moderate hypothermia after severe traumatic brain injury in children. *Neurosurgery* 2005; 56:740–754

3. Agus MSD, Steil GM, Wypij D, et al.: Tight glycemic control versus standard care after pediatric cardiac surgery. *N. Engl. J. Med.* 2012; 367:1208–1219

4. Akinci SB, Kanbak M, Guler A, et al.: Remifentanil versus fentanyl for short-term analgesia-based sedation in mechanically ventilated postoperative children. *Pediatric Anesthesia* 2005; 15:870–878

5. Albers MJ, Steyerberg EW, Hazebroek FW, et al.: Glutamine supplementation of parenteral nutrition does not improve intestinal permeability, nitrogen balance, or outcome in newborns and infants undergoing digestive-tract surgery: results from a double-blind, randomized, controlled trial. *Annals of Surgery* 2005; 241:599

6. Anene O, Meert KL, Uy H, et al.: Dexamethasone for the prevention of postextubation airway obstruction: a prospective, randomized, double-blind, placebo-controlled trial. *Critical Care Medicine* 1996; 24:1666–1669

7. Anton N, Cox PN, Massicotte MP, et al.: Heparin-bonded central venous catheters do not reduce thrombosis in infants with congenital heart disease: a blinded randomized, controlled trial. *PEDIATRICS* 2009; 123:e453–e458

8. Arino M, Barrington JP, Morrison AL, et al.: Management of the changeover of inotrope infusions in children. *Intensive and Critical Care Nursing* 2004; 20:275–280

9. Arnold JH, Hanson JH, Toro-Figuero LO, et al.: Prospective, randomized comparison of high-frequency oscillatory ventilation and conventional mechanical ventilation in pediatric respiratory failure. *Critical Care Medicine* 1994; 22:1530–1539

10. Aydogan MS, Korkmaz MF, Ozgül U, et al.: Pain, fentanyl consumption, and delirium in adolescents after scoliosis surgery: dexmedetomidine vs midazolam. *Pediatric Anesthesia* 2013;

11. Baldasso E, Garcia PCR, Piva JP, et al.: Pilot safety study of low-dose vasopressin in non-septic critically ill children. *Intensive Care Med* 2009; 35:355–359

12. de Barbieri I, Frigo AC, Zampieron A: Quick change versus double pump while changing the infusion of inotropes: an experimental study. *Nurs Crit Care* 2009; 14:200–206

13. Barbosa E, Moreira EAM, Goes JE, et al.: Pilot study with a glutamine-supplemented enteral formula in critically ill infants. *Rev. Hosp. Clin.* 1999; 54:21–24

14. Barret JP, Jeschke MG, Herndon DN: Selective decontamination of the digestive tract in severely burned pediatric patients. *Burns* 2001; 27:439–445

15. Barton P, Garcia J, Kouatli A, et al.: Hemodynamic effects of IV milrinone lactate in pediatric patients with septic shock: a prospective, double-blinded, randomized, placebo-controlled, interventional study. *CHEST* 1996; 109:1302–1312

16. Basnet S, Mander G, Andoh J, et al.: Safety, efficacy, and tolerability of early initiation of noninvasive positive pressure ventilation in pediatric patients admitted with status asthmaticus: a pilot study. *Pediatric Critical Care Medicine* 2012; 13:393–398

17. Behera SK, Zuccaro JC, Wetzel GT, et al.: Nesiritide improves hemodynamics in children with dilated cardiomyopathy: a pilot study. *Pediatr Cardiol* 2008; 30:26–34

18. Behrens R, Hofbeck M, Singer H, et al.: Frequency of stress lesions of the upper gastrointestinal tract in paediatric patients after cardiac surgery: effects of prophylaxis. *Br Heart J* 1994; 72:186–189

19. Berens RJ, Meyer MT, Mikhailov TA, et al.: A prospective evaluation of opioid weaning in opioid-dependent pediatric critical care patients. *Anesth. Analg.* 2006; 102:1045–1050

20. Van den Berghe G, de Zegher F, Lauwers P: Dopamine suppresses pituitary function in infants and children. *Critical Care Medicine* 1994; 22:1747

21. Bettendorf M, Schmidt KG, Grulich-Henn J, et al.: Tri-iodothyronine treatment in children after cardiac surgery: a double-blind, randomised, placebo-controlled study. *The Lancet* 2000; 356:529–534

22. de Betue CT, van Waardenburg DA, Deutz NE, et al.: Increased protein-energy intake promotes anabolism in critically ill infants with viral bronchiolitis: a double-blind randomised controlled trial. *Archives of Disease in Childhood* 2011; 96:817–822

23. Bigham MT, Jacobs BR, Monaco MA, et al.: Helium/oxygen-driven albuterol nebulization in the management of children with status asthmaticus: a randomized, placebo-controlled trial. *Pediatric Critical Care Medicine* 2010; 11:356

24. Bindl L, Buderus S, Ramirez M, et al.: Cisapride reduces postoperative gastrocaecal transit time after cardiac surgery in children. *Intensive Care Med* 1996; 22:977–980

25. Biswas AK, Bruce DA, Sklar FH, et al.: Treatment of acute traumatic brain injury in children with moderate hypothermia improves intracranial hypertension. *Critical Care Medicine* 2002; 30:2742–2751

26. Bogie AL, Towne D, Luckett PM, et al.: Comparison of intravenous terbutaline versus normal saline in pediatric patients on continuous high-dose nebulized albuterol for status asthmaticus. *Pediatr Emerg Care* 2007; 23:355–361

27. Botrán M, López-Herce J, Mencía S, et al.: Enteral nutrition in the critically ill child: comparison of standard and protein-enriched diets. *The Journal of Pediatrics* 2011; 159:27–32.e1

28. Bouvé LR, Rozmus CL, Giordano P: Preparing parents for their child's transfer from the PICU to the pediatric floor. *Appl Nurs Res* 1999; 12:114–120

29. Bowens CD, Thompson JA, Thompson MT, et al.: A trial of methadone tapering schedules in pediatric intensive care unit patients exposed to prolonged sedative infusions. *Pediatric Critical Care Medicine* 2011; 12:504–511

30. Boyer LV, Theodorou AA, Berg RA, et al.: Antivenom for critically ill children with neurotoxicity from scorpion stings. *N. Engl. J. Med.* 2009; 360:2090–2098

31. Briassoulis G, Filippou O, Kanariou M, et al.: Temporal nutritional and inflammatory changes in children with severe head injury fed a regular or an immune-enhancing diet: a randomized, controlled trial. *Pediatric Critical Care Medicine* 2006; 7:56–62

32. Briassoulis G, Filippou O, Hatzi E, et al.: Early enteral administration of immunonutrition in critically ill children: results of a blinded randomized controlled clinical trial. *Nutrition* 2005; 21:799–807

33. Briassoulis G, Filippou O, Kanariou M, et al.: Comparative effects of early randomized immune or non-immune-enhancing enteral nutrition on cytokine production in children with septic shock. *Intensive Care Med* 2005; 31:851–858

34. Broner CW, Stidham GL, Westenkirchner DF, et al.: A prospective, randomized, double-blind comparison of calcium chloride and calcium gluconate therapies for hypocalcemia in critically ill children. *The Journal of Pediatrics* 1990; 117:986–989

35. Brutocao D, Bratton SL, Thomas JR, et al.: Comparison of hetastarch with albumin for postoperative volume expansion in children after cardiopulmonary bypass. *Journal of Cardiothoracic and Vascular Anesthesia* 1996; 10:348–351

36. Buckingham SC, Jafri HS, Bush AJ, et al.: A randomized, double-blind, placebo-controlled trial of dexamethasone in severe respiratory syncytial virus (RSV) infection: effects on RSV quantity and clinical outcome. *J. Infect. Dis.* 2002; 185:1222–1228

37. Burmester M, Mok Q: Randomised controlled trial comparing cisatracurium and vecuronium infusions in a paediatric intensive care unit. *Intensive Care Med* 2005; 31:686–692

38. Butkovic D, Kralik S, Matolic M, et al.: Postoperative analgesia with intravenous fentanyl PCA vs epidural block after thoracoscopic pectus excavatum repair in children. *British Journal of Anaesthesia* 2007; 98:677–681

39. Cai J, Su Z, Shi Z, et al.: Nitric oxide and milrinone: combined effect on pulmonary circulation after fontan-type procedure: a prospective, randomized study. *The Annals of Thoracic Surgery* 2008; 86:882–888

40. Cam BV, Tuan DT, Fonsmark L, et al.: Randomized comparison of oxygen mask treatment vs. nasal continuous positive airway pressure in dengue shock syndrome with acute respiratory failure. *Journal of Tropical Pediatrics* 2002; 48:335–339

41. Cambonie G, Milési C, Fournier-Favre S, et al.: Clinical effects of heliox administration for acute bronchiolitis in young infants. *CHEST* 2006; 129:676–682

42. Canan O, Çelik Y, Çetin İ, et al.: Düzeltici kalp cerrahisi uygulanan doğuştan kalp hastalıklı çocuklarda postoperatif parenteral beslenme desteğinin değerlendirilmesi. *Cocuk Sagligi Ve Hastaliklari Dergisi* 2007; 50:6–11

43. Carcillo JA, Michael Dean J, Holubkov R, et al.: The randomized comparative pediatric critical illness stress-induced immune suppression (CRISIS) prevention trial. *Pediatric Critical Care Medicine* 2012; 13:165–173

44. Carman B, Cahill T, Warden G, et al.: A prospective, randomized comparison of the volume diffusive respirator vs conventional ventilation for ventilation of burned children. *J Burn Care Rehabil* 2002; 23:444–448

45. de Carvalho Onofre PS, da Luz Gonçalves Pedreira M, Peterlini MAS: Placement of peripherally inserted central catheters in children guided by ultrasound: a prospective randomized, and controlled trial. *Pediatric Critical Care Medicine* 2012; 13:e282–e287

46. Ceelie I, de Wildt SN, Van Dijk M, et al.: Effect of intravenous paracetamol on postoperative morphine requirements in neonates and infants undergoing major noncardiac surgery: a randomized controlled trial. *JAMA* 2013; 309:149–154

47. Cesar RG, de Carvalho WB: l-Epinephrine and dexamethasone in postextubation airway obstruction: a prospective, randomized, double-blind placebo-controlled study. *International Journal of Pediatric Otorhinolaryngology* 2009; 73:1639–1643

48. Chiaretti A, Simeone E, Langer A, et al.: [Analgesic efficacy of ketorolac and fentanyl in pediatric intensive care]. *Pediatr Med Chir* 1997; 19:419–424

49. Cholette JM, Powers KS, Alfieris GM, et al.: Transfusion of cell saver salvaged blood in neonates and infants undergoing open heart surgery significantly reduces RBC and coagulant product transfusions and donor exposures: results of a prospective, randomized, clinical trial. *Pediatric Critical Care Medicine* 2013; 14:137–147

50. Cholette JM, Henrichs KF, Alfieris GM, et al.: Washing red blood cells and platelets transfused in cardiac surgery reduces postoperative inflammation and number of transfusions: results of a prospective, randomized, controlled clinical trial. *Pediatric Critical Care Medicine* 2012; 13:290–299

51. Cholette JM, Rubenstein JS, Alfieris GM, et al.: Children with single-ventricle physiology do not benefit from higher

hemoglobin levels post cavopulmonary connection: results of a prospective, randomized, controlled trial of a restrictive versus liberal red-cell transfusion strategy. *Pediatric Critical Care Medicine* 2011; 12:39–45

52. Choong K, Arora S, Cheng J, et al.: Hypotonic versus isotonic maintenance fluids after surgery for children: a randomized controlled trial. *PEDIATRICS* 2011; 128:857–866

53. Choong K, Bohn D, Fraser DD, et al.: Vasopressin in pediatric vasodilatory shock: a multicenter randomized controlled trial. *American Journal of Respiratory and Critical Care Medicine* 2009; 180:632–639

54. Chopra A, Kumar V, Dutta A: Hypertonic versus normal saline as initial fluid bolus in pediatric septic shock. *Indian J Pediatr* 2011; 78:833–837

55. Chowdhury D, Ojamaa K, Parnell VA, et al.: A prospective randomized clinical study of thyroid hormone treatment after operations for complex congenital heart disease. *J. Thorac. Cardiovasc. Surg.* 2001; 122:1023–1025

56. Cifra HL, Velasco J: A comparative study of the efficacy of 6% Haes-Steril and Ringer's lactate in the management of dengue shock syndrome. *Critical Care And Shock* 2003; 6:95–100

57. Clancy RR, McGaurn SA, Goin JE, et al.: Allopurinol neurocardiac protection trial in infants undergoing heart surgery using deep hypothermic circulatory arrest. *PEDIATRICS* 2001; 108:61–70

58. Coulthard MG, Long DA, Ullman AJ, et al.: A randomised controlled trial of Hartmann's solution versus half normal saline in postoperative paediatric spinal instrumentation and craniotomy patients. *Archives of Disease in Childhood* 2012; 97:491–496

59. Curley MAQ, Hibberd PL, Fineman LD, et al.: Effect of prone positioning on clinical outcomes in children with acute lung injury: a randomized controlled trial. *JAMA* 2005; 294:229–237

60. Darnell CM, Thompson J, Stromberg D, et al.: Effect of low-dose naloxone infusion on fentanyl requirements in critically ill children. *PEDIATRICS* 2008; 121:e1363–71

61. Day RW, Guarín M, Lynch JM, et al.: Inhaled nitric oxide in children with severe lung disease: results of acute and prolonged therapy with two concentrations. *Critical Care Medicine* 1996; 24:215–221

62. Day RW, Allen EM, Witte MK: A randomized, controlled study of the 1-hour and 24-hour effects of inhaled nitric oxide therapy in children with acute hypoxemic respiratory failure. *CHEST* 1997; 112:1324–1331

63. Van Dijk M, Bouwmeester NJ, Duivenvoorden HJ, et al.: Efficacy of continuous versus intermittent morphine administration after major surgery in 0-3-year-old infants; a double-blind randomized controlled trial. *Pain* 2002; 98:305–313

64. Dobyns EL, Cornfield DN, Anas NG, et al.: Multicenter randomized controlled trial of the effects of inhaled nitric oxide therapy on gas exchange in children with acute hypoxemic respiratory failure. *The Journal of Pediatrics* 1999; 134:406

65. Dung NM, Day NP, Tam DT, et al.: Fluid replacement in dengue shock syndrome: a randomized, double-blind comparison of four intravenous-fluid regimens. *Clin. Infect. Dis.* 1999; 29:787–794

66. Eddleston JM, Booker PD, Green JR: Use of ranitidine in children undergoing cardiopulmonary bypass. *Critical Care Medicine* 1989; 17:26–29

67. El-Bayoumi MA, El-Refaey AM, Abdelkader AM, et al.: Comparison of intravenous immunoglobulin and plasma exchange in treatment of mechanically ventilated children with Guillain Barré syndrome: a randomized study. *Critical Care* 2011; 15:R164

68. Fallah R, Gofrani M: Comparison of intravenous lidocaine and midazolam infusion for refractory convulsive status epilepticus in children. *Journal of Pediatric Neurology* 2007; 5:287–290

69. Fanconi S, Klöti J, Meuli M, et al.: Dexamethasone therapy and endogenous cortisol production in severe pediatric head injury. *Intensive Care Med* 1988; 14:163–166

70. Farias JA, Retta A, Alía I, et al.: A comparison of two methods to perform a breathing trial before extubation in pediatric intensive care patients. *Intensive Care Med* 2001; 27:1649–1654

71. Fayyazi A, Karimzadeh P, Torabian S, et al.: Comparison of intravenous midazolam drip with intermittent intravenous diazepam in the treatment of refractory serial seizures in children. *Iranian Journal of Child Neurology* 2012; 6:15–19

72. Fernandes IC, Fernandes JC, Cordeiro A, et al.: [Efficacy and safety of nebulized L-epinephrine associated with dexamethasone in postintubation laringitis]. *J Pediatr (Rio J)* 2001; 77:179–188

73. Foronda FK, Troster EJ, Farias JA, et al.: The impact of daily evaluation and spontaneous breathing test on the duration of pediatric mechanical ventilation: a randomized controlled trial. *Critical Care Medicine* 2011; 39:2526–2533

74. Fraisse A, Butrous G, Taylor MB, et al.: Intravenous sildenafil for postoperative pulmonary hypertension in children with congenital heart disease. *Intensive Care Med* 2010; 37:502–509

75. Fram RY, Cree MG, Wolfe RR, et al.: Intensive insulin therapy improves insulin sensitivity and mitochondrial function in severely burned children. *Critical Care Medicine* 2010; 38:1475–1483

76. Geukers VG, Li Z, Ackermans MT, et al.: High-carbohydrate/low-protein-induced hyperinsulinemia does not improve protein balance in children after cardiac surgery. *Nutrition* 2012; 28:644–650

77. Gharpure V, Meert KL, Sarnaik AP: Efficacy of erythromycin for postpyloric placement of feeding tubes in critically ill children: a randomized, double-blind, placebo controlled study. *JPEN J Parenter Enteral Nutr* 2001; 25:160–165

78. Gooding AM, Bastian JF, Peterson BM, et al.: Safety and efficacy of intravenous immunoglobulin prophylaxis in pediatric head trauma patients: a double-blind controlled trial. *J Crit Care* 1993; 8:212–216

79. Gottschlich MM, Jenkins ME, Mayes T, et al.: An evaluation of the safety of early vs delayed enteral support and effects on clinical, nutritional, and endocrine outcomes after severe burns. *J Burn Care Rehabil* 2002; 23:401–415

80. Greenhalgh DG, Housinger TA, Kagan RJ, et al.: Maintenance of serum albumin levels in pediatric burn patients: a prospective, randomized trial. *The Journal of Trauma: Injury, Infection, and Critical Care* 1995; 39:67–73

81. Greissman A, Silver P, Nimkoff L, et al.: Albumin bolus administration versus continuous infusion in critically ill hypoalbuminemic pediatric patients. *Intensive Care Med* 1996; 22:495–499

82. Guerguerian AM, Gauthier M, Lebel MH, et al.: Ribavirin in ventilated respiratory syncytial virus bronchiolitis a randomized, placebo-controlled trial. *American Journal of Respiratory and Critical Care Medicine* 1999; 160:829–834

83. Gupta A, Daggett C, Drant S, et al.: Prospective randomized trial of ketorolac after congenital heart surgery. *Journal of Cardiothoracic and Vascular Anesthesia* 2004; 18:454–457

84. Gupta K, Gupta VK, Muralindharan J, et al.: Randomized controlled trial of interrupted versus continuous sedative infusions in ventilated children. *Pediatric Critical Care Medicine* 2012; 13:131–135

85. Harel Y, Vardi A, Quigley R, et al.: Extubation failure due to post-extubation stridor is better correlated with neurologic impairment than with upper airway lesions in critically ill pediatric patients. *International Journal of Pediatric Otorhinolaryngology* 1997; 39:147–158

86. Hatem TP, Lira PIC, Mattos SS: The therapeutic effects of music in children following cardiac surgery. *J Pediatr (Rio J)* 2006; 82:186–192

87. Heulitt MJ, Farrington EA, O'Shea TM, et al.: Double-blind, randomized, controlled trial of papaverine-containing infusions to prevent failure of arterial catheters in pediatric patients. *Critical Care Medicine* 1993; 21:825–829

88. Hoffman TM, Wernovsky G, Atz AM, et al.: Efficacy and safety of milrinone in preventing low cardiac output syndrome in infants and children after corrective surgery for congenital heart disease. *Circulation* 2003; 107:996–1002

89. Honeycutt TCB, Khashab El M, Wardrop RM III, et al.: Probiotic administration and the incidence of nosocomial infection in pediatric intensive care: A randomized placebo-controlled trial. *Pediatric Critical Care Medicine* 2007; 8:452–458

90. Horn D, Chaboyer W: Gastric feeding in critically ill children: a randomized controlled trial. *Am. J. Crit. Care* 2003; 12:461–468

91. Hsin SH, Fontes AM, Bousso A, et al.: Terapêutica de crianças com sepse de apresentação purpúrica com dois esquemas antibióticos. *Jornal de Pediatria, Rio de Janeiro* 1998; 74:S315–S324

92. Hutchison JS, Ward RE, Lacroix J, et al.: Hypothermia therapy after traumatic brain injury in children. *N. Engl. J. Med.* 2008; 358:2447–2456

93. Ibrahim TS, El-Mohamady HS: Inhaled nitric oxide and prone position: how far they can improve oxygenation in pediatric patients with acute respiratory distress syndrome? *J. Med. Sci* 2007; 7:390–395

94. Jaarsma AS, Knoester H, van Rooyen F, et al.: Biphasic positive airway pressure ventilation (PeV+) in children. *Critical Care* 2001; 5:174–177

95. Jack T, Boehne M, Brent BE, et al.: In-line filtration reduces severe complications and length of stay on pediatric intensive care unit: a prospective, randomized, controlled trial. *Intensive Care Med* 2012; 38:1008–1016

96. Jacobs BR, Barr LL, Brilli RJ, et al.: Intracatheter nitroglycerin infusion fails to prevent catheter-related venous thrombosis: a randomized, controlled trial. *Intensive Care Med* 2001; 27:187–192

97. Jacobs BR, Nadkarni V, Goldstein B, et al.: Nutritional immunomodulation in critically ill children with acute lung injury: feasibility and impact on circulating biomarkers. *Pediatric Critical Care Medicine* 2013; 14:e45–56

98. Jacobs BR, Lyons K, Brilli RJ: Erythropoietin therapy in children with bronchiolitis and anemia. *Pediatric Critical Care Medicine* 2003; 4:44–48

99. Jácomo ADN, Carmona F, Matsuno AK, et al.: Effect of oral hygiene with 0.12% chlorhexidine gluconate on the incidence of nosocomial pneumonia in children undergoing cardiac surgery. *Infect Control Hosp Epidemiol* 2011; 32:591–596

100. Jeschke MG, Kulp GA, Kraft R, et al.: Intensive insulin therapy in severely burned pediatric patients: a prospective randomized trial. *American Journal of Respiratory and Critical Care Medicine* 2010; 182:351–359

101. Jeschke MG, Barrow RE, Herndon DN: Recombinant human growth hormone treatment in pediatric burn patients and its role during the hepatic acute phase response. *Critical Care Medicine* 2000; 28:1578–1584

102. Jeschke MG, Finnerty CC, Kulp GA, et al.: Combination of recombinant human growth hormone and propranolol decreases hypermetabolism and inflammation in severely burned children. *Pediatric Critical Care Medicine* 2008; 9:209–216

103. Jeschke MG, Finnerty CC, Suman OE, et al.: The effect of oxandrolone on the endocrinologic, inflammatory, and hypermetabolic responses during the acute phase postburn. *Annals of Surgery* 2007; 246:351–362

104. Jeschke MG, Norbury WB, Finnerty CC, et al.: Propranolol does not increase inflammation, sepsis, or infectious episodes in severely burned children. *The Journal of Trauma: Injury, Infection, and Critical Care* 2007; 62:676–681

105. Jouvet PA, Payen V, Gauvin F, et al.: Weaning children from mechanical ventilation with a computer-driven protocol: a pilot trial. *Intensive Care Med* 2013; 39:919–925

106. Kamat P, Favaloro-Sabatier J, Rogers K, et al.: Use of methylene blue spectrophotometry to detect subclinical aspiration in enterally fed intubated pediatric patients. *Pediatric Critical Care Medicine* 2008; 9:299–303

107. de Kleijn ED, de Groot R, Hack CE, et al.: Activation of protein C following infusion of protein C concentrate in children with severe meningococcal sepsis and purpura fulminans: a randomized, double-blinded, placebo-controlled, dose-finding study. *Critical Care Medicine* 2003; 31:1839–1847

108. Klein BS, Perloff WH, Maki DG: Reduction of nosocomial infection during pediatric intensive care by protective isolation. *N. Engl. J. Med.* 1989; 320:1714–1721

109. Klein SM, Hauser GJ, Anderson BD, et al.: Comparison of intermittent versus continuous infusion of propofol for elective oncology procedures in children. *Pediatric Critical Care Medicine* 2003; 4:78–82

110. Kline AM, Sorce L, Sullivan C, et al.: Use of a noninvasive electromagnetic device to place transpyloric feeding tubes in critically ill children. *Am. J. Crit. Care* 2011; 20:453–460

111. Klinge JM, Scharf J, Hofbeck M, et al.: Intermittent administration of furosemide versus continuous infusion in the postoperative management of children following open heart surgery. *Intensive Care Med* 1997; 23:693–697

112. Krafte-Jacobs B, Persinger M, Carver J, et al.: Rapid placement of transpyloric feeding tubes: a comparison of pH-assisted and standard insertion techniques in children. *PEDIATRICS* 1996; 98:242–248

113. Kumar S, Bansal A, Chakrabarti A, et al.: Evaluation of efficacy of probiotics in prevention of candida colonization in a PICU-a randomized controlled trial. *Critical Care Medicine* 2013; 41:565–572

114. Kusahara DM, Friedlander LT, Peterlini MAS, et al.: Oral care and oropharyngeal and tracheal colonization by Gram-negative pathogens in children. *Nurs Crit Care* 2012; 17:115–122

115. Lacroix J, Infante-Rivard C, Gauthier M, et al.: Upper gastrointestinal tract bleeding acquired in a pediatric intensive care unit: prophylaxis trial with cimetidine. *The Journal of Pediatrics* 1986; 108:1015–1018

116. Lacroix J, Hébert PC, Hutchison JS, et al.: Transfusion strategies for patients in pediatric intensive care units. *N. Engl. J. Med.* 2007; 356:1609–1619

117. Laitinen P, Happonen JM, Sairanen H, et al.: Amrinone versus dopamine-nitroglycerin after reconstructive surgery for complete atrioventricular septal defect. *Journal of Cardiothoracic and Vascular Anesthesia* 1997; 11:870–874

118. Larsen BMK, Goonewardene LA, Joffe AR, et al.: Pre-treatment with an intravenous lipid emulsion containing fish oil (eicosapentaenoic and docosahexaenoic acid) decreases inflammatory markers after open-heart surgery in infants: a randomized, controlled trial. *Clinical Nutrition* 2012; 31:322–329

119. Lechner E, Hofer A, Leitner-Peneder G, et al.: Levosimendan versus milrinone in neonates and infants after corrective open-heart surgery: a pilot study. *Pediatric Critical Care Medicine* 2012; 13:542–548

120. Leenen FH, Balfe JA, Pelech AN, et al.: Postoperative hypertension after repair of coarctation of aorta in children: protective effect of propranolol? *Am. Heart J.* 1987; 113:1164–1173

121. Lenz AM, Vassallo JC, Moreno GE, et al.: [Prevention of catheter-related infection: usefulness and cost-effectiveness of antiseptic catheters in children]. *Arch Argent Pediatr* 2010; 108:209–215

122. Levy I, Katz J, Solter E, et al.: Chlorhexidine-impregnated dressing for prevention of colonization of central venous catheters in infants and children: a randomized controlled study. *The Pediatric Infectious Disease Journal* 2005; 24:676–679

123. Li H, Lu G, Shi W, et al.: Protective effect of moderate hypothermia on severe traumatic brain injury in children. *J. Neurotrauma* 2009; 26:1905–1909

124. Li Z-P, Cao Q, Xing Q-S: [Effect of milkvetch injection on cardiac function and hemodynamics in children with tetralogy of Fallot after radical operation]. *Zhongguo Zhong Xi Yi Jie He Za Zhi* 2003; 23:891–894

125. Lieh-Lai MW, Kauffman RE, Uy HG, et al.: A randomized comparison of ketorolac tromethamine and morphine for postoperative analgesia in critically ill children. *Critical Care Medicine* 1999; 27:2786–2791

126. Liet J-M, Millotte B, Tucci M, et al.: Noninvasive therapy with helium–oxygen for severe bronchiolitis. *The Journal of Pediatrics* 2005; 147:812–817

## Appendix B: Included Trials


127. Lindsay CA, Barton P, Lawless S, et al.: Pharmacokinetics and pharmacodynamics of milrinone lactate in pediatric patients with septic shock. *The Journal of Pediatrics* 1998; 132:329–334

128. Liu M, Zheng P, Zheng X-Y, et al.: [Rational control on postoperative blood glucose levels in infants with congenital heart disease]. *Zhongguo Wei Zhong Bing Ji Jiu Yi Xue* 2012; 24:244–246

129. López-Herce J, Dorao P, Elola P, et al.: Frequency and prophylaxis of upper gastrointestinal hemorrhage in critically ill children: a prospective study comparing the efficacy of almagate, ranitidine, and sucralfate. *Critical Care Medicine* 1992; 20:1082-1089

130. López-Herce J, Velasco J, Codoceo R, et al.: Ranitidine prophylaxis in acute gastric mucosal damage in critically ill pediatric patients. *Critical Care Medicine* 1988; 16:591–593

131. Loukanov T, Bucsenez D, Springer W, et al.: Comparison of inhaled nitric oxide with aerosolized iloprost for treatment of pulmonary hypertension in children after cardiopulmonary bypass surgery. *Clin Res Cardiol* 2011; 100:595–602

132. Luchetti M, Casiraghi G, Valsecchi R, et al.: Porcine-derived surfactant treatment of severe bronchiolitis. *Acta Anaesthesiol Scand* 1998; 42:805–810

133. Luchetti M, Ferrero F, Gallini C, et al.: Multicenter, randomized, controlled study of porcine surfactant in severe respiratory syncytial virus-induced respiratory failure. *Pediatric Critical Care Medicine* 2002; 3:261–268

134. Luciani GB, Nichani S, Chang AC, et al.: Continuous versus intermittent furosemide infusion in critically ill infants after open heart operations. *The Annals of Thoracic Surgery* 1997; 64:1133–1139

135. Lukas JC, Karikas G, Gazouli M, et al.: Pharmacokinetics of teicoplanin in an ICU population of children and infants. *Pharmaceutical research* 2004; 21:2064–2071

136. Lyons KA, Brilli RJ, Wieman RA, et al.: Continuation of transpyloric feeding during weaning of mechanical ventilation and tracheal extubation in children: a randomized controlled trial. *JPEN J Parenter Enteral Nutr* 2002; 26:209–213

137. Macnab AJ, Levine M, Glick N, et al.: Midazolam following open heart surgery in children: haemodynamic effects of a loading dose. *Paediatr Anaesth* 1996; 6:387–397

138. Malhotra D, Gurcoo S, Qazi S, et al.: Randomized comparative efficacy of dexamethasone to prevent postextubation upper airway complications in children and adults in ICU. *Indian J Anaesth* 2009; 53:442–449

139. Malley R, DeVincenzo J, Ramilo O, et al.: Reduction of respiratory syncytial virus (RSV) in tracheal aspirates in intubated infants by use of humanized monoclonal antibody to RSV F protein. *J. Infect. Dis.* 1998; 178:1555–1561

140. Marik PE, Havlik I, Monteagudo FS, et al.: The pharmacokinetic of amikacin in critically ill adult and paediatric patients: comparison of once- versus twice-daily dosing regimens. *J. Antimicrob. Chemother.* 1991; 27 Suppl C:81–89

141. Marin VB, Rodriguez-Osiac L, Schlessinger L, et al.: Controlled study of enteral arginine supplementation in burned children: impact on immunologic and metabolic status. *Nutrition* 2006; 22:705–712

142. Marraro GA, Luchetti M, Spada C, et al.: Selective medicated (normal saline and exogenous surfactant) bronchoalveolar lavage in severe aspiration syndrome in children. *Pediatric Critical Care Medicine* 2007; 8:476–481

143. Mecott GA, Herndon DN, Kulp GA, et al.: The use of exenatide in severely burned pediatric patients. *Critical Care* 2010; 14:R153

144. Meert KL, Sarnaik AP, Gelmini MJ, et al.: Aerosolized ribavirin in mechanically ventilated children with respiratory syncytial virus lower respiratory tract disease: a prospective, double-blind, randomized trial. *Critical Care Medicine* 1994; 22:566–572

145. Meert KL, Daphtary KM, Metheny NA: Gastric vs small-bowel feeding in critically ill children receiving mechanical ventilation: a randomized controlled trial. *CHEST* 2004; 126:872–878

146. Mehta V, Singhi P, Singhi S: Intravenous sodium valproate versus diazepam infusion for the control of refractory status epilepticus in children: a randomized controlled trial. *Journal of Child Neurology* 2007; 22:1191–1197

147. Melchers P, Maluck A, Suhr L, et al.: An early onset rehabilitation program for children and adolescents after traumatic brain injury (TBI): methods and first results. *Restor. Neurol. Neurosci.* 1999; 14:153–160

148. Melnyk BM, Alpert-Gillis LJ, Hensel PB, et al.: Helping mothers cope with a critically ill child: a pilot test of the COPE intervention. *Res Nurs Health* 1997; 20:3–14

149. Milési C, Matecki S, Jaber S, et al.: 6 cmH 2O continuous positive airway pressure versus conventional oxygen therapy in severe viral bronchiolitis: A randomized trial. *Pediatr. Pulmonol.* 2012; 48:45–51

150. Miller OI, Tang SF, Keech A, et al.: Inhaled nitric oxide and prevention of pulmonary hypertension after congenital heart surgery: a randomised double-blind study. *The Lancet* 2000; 356:1464–1469

151. Milstone AM, Elward A, Song X, et al.: Daily chlorhexidine bathing to reduce bacteraemia in critically ill children: a multicentre, cluster-randomised, crossover trial. *The Lancet* 2013;

152. Möller JC, Schaible T, Roll C, et al.: Treatment with bovine surfactant in severe acute respiratory distress syndrome in children: a randomized multicenter study. *Intensive Care Med* 2003; 29:437–446

153. Molon M, Piva J, Karcher P, et al.: Clonidina associada à morfina e midazolam em crianças submetidas à ventilação mecânica: estudo aleatório, duplamente encoberto e placebo controlado. *Rev Bras Ter Intensiva* 2007; 19:284–291

154. Momeni M, Rubay J, Matta A, et al.: Levosimendan in congenital cardiac surgery: a randomized, double-blind clinical trial. *Journal of Cardiothoracic and Vascular Anesthesia* 2011; 25:419–424

155. Mondal RK, Singhi SC, Chakrabarti A, et al.: Randomized comparison between fluconazole and itraconazole for the treatment of candidemia in a pediatric intensive care unit: a preliminary study. *Pediatric Critical Care Medicine* 2004; 5:561–565

156. Montañana PÁ, Modesto i Alapont V, Ocón AP, et al.: The use of isotonic fluid as maintenance therapy prevents iatrogenic hyponatremia in pediatrics: a randomized, controlled open study. *Pediatric Critical Care Medicine* 2008; 9:589–597

157. Moraes MA de, Bonatto RC, Carpi MF, et al.: Comparison between intermittent mandatory ventilation and synchronized intermittent mandatory ventilation with pressure support in children. *J Pediatr (Rio J)* 2008; 0

158. Morrow B, Futter M, Argent A: A recruitment manoeuvre performed after endotracheal suction does not increase dynamic compliance in ventilated paediatric patients: a randomised controlled trial. *Australian Journal of Physiotherapy* 2007; 53:163

159. Nadel S, Goldstein B, Williams MD, et al.: Drotrecogin alfa (activated) in children with severe sepsis: a multicentre phase III randomised controlled trial. *Lancet* 2007; 369:836–843

160. Nahum E, Levy I, Katz J, et al.: Efficacy of subcutaneous tunneling for prevention of bacterial colonization of femoral central venous catheters in critically ill children. *The Pediatric Infectious Disease Journal* 2002; 21:1000–1004

161. Namachivayam P, Theilen U, Butt WW, et al.: Sildenafil prevents rebound pulmonary hypertension after withdrawal of nitric oxide in children. *American Journal of Respiratory and Critical Care Medicine* 2006; 174:1042–1047

162. Natale JE, Guerguerian A-M, Joseph JG, et al.: Pilot study to determine the hemodynamic safety and feasibility of magnesium sulfate infusion in children with severe traumatic brain injury. *Pediatric Critical Care Medicine* 2007; 8:1–9

163. de Neef M, Heijboer H, van Woensel JBM, et al.: The efficacy of heparinization in prolonging patency of arterial and central venous catheters in children: a randomized double-blind trial. *Pediatr Hematol Oncol* 2002; 19:553–560

164. Nguyen TC, Han YY, Kiss JE, et al.: Intensive plasma exchange increases a disintegrin and metalloprotease with thrombospondin motifs-13 activity and reverses organ dysfunction in children with thrombocytopenia-associated multiple organ failure. *Critical Care Medicine* 2008; 36:2878–2887

165. Nutman J, Brooks LJ, Deakins KM, et al.: Racemic versus l-epinephrine aerosol in the treatment of postextubation laryngeal edema: results from a prospective, randomized, double-blind study. *Critical Care Medicine* 1994; 22:1591–1594

166. Oliveira CF, Oliveira DSF, Gottschald AFC, et al.: ACCM/PALS haemodynamic support guidelines for paediatric septic shock: an outcomes comparison with and without monitoring central venous oxygen saturation. *Intensive Care Med* 2008; 34:1065–1075

167. Papo MC, Frank J, Thompson AE: A prospective, randomized study of continuous versus intermittent nebulized albuterol for severe status asthmaticus in children. *Critical Care Medicine* 1993; 21:1479–1486

168. Parkinson L, Hughes J, Gill A, et al.: A randomized controlled trial of sedation in the critically ill. *Paediatr Anaesth* 1997; 7:405–410

169. Pedreira MLG, Kusahara DM, de Carvalho WB, et al.: Oral care interventions and oropharyngeal colonization in children receiving mechanical ventilation. *Am. J. Crit. Care* 2009; 18:319–328

170. Pemberton VL, Browning B, Webster A, et al.: Therapeutic hypothermia after pediatric cardiac arrest trials: the vanguard phase experience and implications for other trials. *Pediatric Critical Care Medicine* 2013; 14:19–26

171. Perondi MBM, Reis AG, Paiva EF, et al.: A comparison of high-dose and standard-dose epinephrine in children with cardiac arrest. *N. Engl. J. Med.* 2004; 350:1722–1730

172. Phipps LM, Weber MD, Ginder BR, et al.: A randomized controlled trial comparing three different techniques of nasojejunal feeding tube placement in critically ill children. *JPEN J Parenter Enteral Nutr* 2005; 29:420–424

173. Pierce CM, Wade A, Mok Q: Heparin-bonded central venous lines reduce thrombotic and infective complications in critically ill children. *Intensive Care Med* 2000; 26:967–972

174. Prabhakaran P, Reddy AT, Oakes WJ, et al.: A pilot trial comparing cerebral perfusion pressure-targeted therapy to intracranial pressure-targeted therapy in children with severe traumatic brain injury. *J. Neurosurg.* 2004; 100:454–459

175. Prasad SR, Simha P, Jagadeesh AM: Comparative study between dexmedetomidine and fentanyl for sedation during mechanical ventilation in post-operative paediatric cardiac surgical patients. *Indian J Anaesth* 2012; 56:547

176. Preutthipan A, Poomthavorn P, Sumanapisan A, et al.: A prospective, randomized double-blind study in children comparing two doses of nebulized l-epinephrine in postintubation croup. *J Med Assoc Thai* 2005; 88:508–512

177. Prins SA, Van Dijk M, van Leeuwen P, et al.: Pharmacokinetics and analgesic effects of intravenous propacetamol vs rectal paracetamol in children after major craniofacial surgery. *Pediatric Anesthesia* 2008; 18:582–592

178. Randolph AG, Wypij D, Venkataraman ST, et al.: Effect of mechanical ventilator weaning protocols on respiratory outcomes in infants and children. *JAMA* 2002; 288:2561–2568

179. Ream RS, Loftis LL, Albers GM, et al.: Efficacy of IV theophylline in children with severe status asthmaticus. *CHEST* 2001; 119:1480–1488

180. Ream RS, Hauver JF, Lynch RE, et al.: Low-dose inhaled nitric oxide improves the oxygenation and ventilation of infants and children with acute, hypoxemic respiratory failure. *Critical Care Medicine* 1999; 27:989–996

181. Reeves JH, Butt WW, Shann F, et al.: Continuous plasmafiltration in sepsis syndrome. *Critical Care Medicine* 1999; 27:2096–2104

182. Rey C, Los-Arcos M, Hernández A, et al.: Hypotonic versus isotonic maintenance fluids in critically ill children: a multicenter prospective randomized study. *Acta Paediatrica* 2011; 100:1138–1143

183. Ricci Z, Garisto C, Favia I, et al.: Levosimendan infusion in newborns after corrective surgery for congenital heart disease: randomized controlled trial. *Intensive Care Med* 2012; 38:1198–1204

184. Ridling DA, Martin LD, Bratton SL: Endotracheal suctioning with or without instillation of isotonic sodium chloride solution in critically ill children. *Am. J. Crit. Care* 2003; 12:212–219

185. Riethmueller J, Borth-Bruhns T, Kumpf M, et al.: Recombinant human deoxyribonuclease shortens ventilation time in young, mechanically ventilated children. *Pediatr. Pulmonol.* 2005; 41:61–66

186. Rodríguez JA, Dessauer Von B, Duffau G: Utilización de la CPAP de forma no invasiva en la laringitis postextubación del paciente pediátrico. Estudio controlado y aleatorizado. *Arch Bronconeumol* 2002; 38:463–467

187. Rouine-Rapp K, Mello DM, Hanley FL, et al.: Effect of enalaprilat on postoperative hypertension after surgical repair of coarctation of the aorta. *Pediatric Critical Care Medicine* 2003; 4:327–332

188. Rushforth K: A randomised controlled trial of weaning from mechanical ventilation in paediatric intensive care (PIC). Methodological and practical issues. *Intensive Crit Care Nurs* 2005; 21:76–86

189. Ruza F, Alvarado F, Herruzo R, et al.: Prevention of nosocomial infection in a pediatric intensive care unit (PICU) through the use of selective digestive decontamination. *Eur. J. Epidemiol.* 1998; 14:719–727

190. Sakellaris G, Kotsiou M, Tamiolaki M, et al.: Prevention of complications related to traumatic brain injury in children and adolescents with creatine administration: an open label randomized pilot study. *The Journal of Trauma: Injury, Infection, and Critical Care* 2006; 61:322–329

191. Samransamruajkit R, Jirapaiboonsuk S, Siritantiwat S, et al.: Effect of frequency of ventilator circuit changes (3 vs 7 days) on the rate of ventilator-associated pneumonia in PICU. *J Crit Care* 2010; 25:56–61

192. Santana JC, Barreto SSM, Piva JP, et al.: Estudo controlado do uso endovenoso de sulfato de magnésio ou de salbutamol no tratamento precoce da crise de asma aguda grave em crianças. *J Pediatr (Rio J)* 2001; 77:279–287

193. Saul JP, Scott WA, Brown S, et al.: Intravenous amiodarone for incessant tachyarrhythmias in children: a randomized, double-blind, antiarrhythmic drug trial. *Circulation* 2005; 112:3470–3477

194. Schroeder AR, Axelrod DM, Silverman NH, et al.: A continuous heparin infusion does not prevent catheter-related thrombosis in infants after cardiac surgery. *Pediatric Critical Care Medicine* 2010; 11:489–495

195. Schroth M, Plank C, Meissner U, et al.: Hypertonic-hyperoncotic solutions improve cardiac function in children after open-heart surgery. *PEDIATRICS* 2006; 118:e76–e84

196. Schultz TR, Lin RJ, Watzman HM, et al.: Weaning children from mechanical ventilation: a prospective randomized trial of protocol-directed versus physician-directed weaning. *Respiratory care* 2001; 46:772

197. Scoble MK, Copnell B, Taylor A, et al.: Effect of reusing suction catheters on the occurrence of pneumonia in children. *Heart Lung* 2001; 30:225–233

198. Sebastian MR, Lodha R, Kapil A, et al.: Oral mucosal decontamination with chlorhexidine for the prevention of ventilator-associated pneumonia in children—a randomized, controlled trial. *Pediatric Critical Care Medicine* 2012; 13:e305–e310

199. Semsroth M, Hiesmayr M: [Continuous application of morphine is more effective than a bolus administration in the postoperative analgesia and sedation of children]. *Anaesthesist* 1990; 39:552–556

200. Shahid SK: Efficacy and safety of cefepime in late-onset ventilator-associated pneumonia in infants: a pilot randomized and controlled study. *Ann. Trop. Med. Parasitol.* 2008; 102:63–71

201. da Silva P, Paulo C, de Oliveira Iglesias S, et al.: Bedside transpyloric tube placement in the pediatric intensive care unit: a modified insufflation air technique. *Intensive Care Med* 2002; 28:943–946

202. Da Silva PSL, Iglesias SBO, Leão FVF, et al.: Procedural sedation for insertion of central venous catheters in children: comparison

of midazolam/fentanyl with midazolam/ketamine. *Pediatric Anesthesia* 2007; 17:358–363

203. Silva PSL, Fonseca MCM, Iglesias SBO, et al.: Nebulized 0.5, 2.5 and 5 mL l-epinephrine for post-extubation stridor in children: a prospective, randomized, double-blind clinical trial. *Intensive Care Med* 2011; 38:286–293

204. Simakachorn N, Bibiloni R, Yimyaem P, et al.: Tolerance, safety, and effect on the faecal microbiota of an enteral formula supplemented with pre- and probiotics in critically ill children. *Journal of Pediatric Gastroenterology and Nutrition* 2011; 53:174–181

205. Simma B, Burger R, Falk M, et al.: A prospective, randomized, and controlled study of fluid management in children with severe head injury: lactated Ringer's solution versus hypertonic saline. *Critical Care Medicine* 1998; 26:1265–1270

206. Singh NC, Kissoon N, Mofada al S, et al.: Comparison of continuous versus intermittent furosemide administration in postoperative pediatric cardiac patients. *Critical Care Medicine* 1992; 20:17–21

207. Singhi S, Järvinen A, Peltola H: Increase in serum osmolality is possible mechanism for the beneficial effects of glycerol in childhood bacterial meningitis. *The Pediatric Infectious Disease Journal* 2008; 27:892–896

208. Singhi S, Murthy A, Singhi P, et al.: Continuous midazolam versus diazepam infusion for refractory convulsive status epilepticus. *Journal of Child Neurology* 2002; 17:106–110

209. Sinha A, Jayashree M, Singhi S: Aerosolized l-epinephrine vs budesonide for post extubation stridor: a randomized controlled trial. *Indian Pediatr* 2010; 47:317–322

210. Slota M, Green M, Farley A, et al.: The role of gown and glove isolation and strict handwashing in the reduction of nosocomial infection in children with solid organ transplantation. *Critical Care Medicine* 2001; 29:405–412

211. Smith DW, Frankel LR, Mathers LH, et al.: A controlled trial of aerosolized ribavirin in infants receiving mechanical ventilation for severe respiratory syncytial virus infection. *N. Engl. J. Med.* 1991; 325:24–29

212. Smith SD, Jackson RJ, Hannakan CJ, et al.: Selective decontamination in pediatric liver transplants. A randomized prospective study. *Transplantation* 1993; 55:1306

213. Solana MJ, López-Herce J, Botrán M, et al.: Efectos hemodinámicos del omeprazol por vía intravenosa en niños en estado crítico. *Anales de Pediatría* 2013; 78:167–172

214. Soler M, Raszynski A, Kandrotas RJ, et al.: Fewer interventions in the immediate post-extubation management of pediatric intensive care unit patients: safety and cost containment. *J Crit Care* 1997; 12:173–176

215. Sorce LR, Hamilton SM, Gauvreau K, et al.: Preventing corneal abrasions in critically ill children receiving neuromuscular blockade: a randomized, controlled trial. *Pediatric Critical Care Medicine* 2009; 10:171–175

216. Spalding HK, Sullivan KJ, Soremi O, et al.: Bedside placement of transpyloric feeding tubes in the pediatric intensive care unit using gastric insufflation. *Critical Care Medicine* 2000; 28:2041–2044

217. Srinivasan R, Meyer R, Padmanabhan R, et al.: Clinical safety of *Lactobacillus casei* shirota as probiotic in critically ill children. *Journal of Pediatric Gastroenterology and Nutrition* 2006; 42:171–173

218. Stocker C, Penny DJ, Brizard CP, et al.: Intravenous sildenafil and inhaled nitric oxide: a randomised trial in infants after cardiac surgery. *Intensive Care Med* 2003; 29:1996–2003

219. Tan LL, Huang JF, Wang H: [The effect of rehabilitation training on postoperative recovery of children with congenital heart disease]. *Zhonghua Hu Li Za Zhi* 1996; 31:314–315

220. Tellez DW, Galvis AG, Storgion SA, et al.: Dexamethasone in the prevention of postextubation stridor in children. *The Journal of Pediatrics* 1991; 118:289–294

221. Thomas NJ, Guardia CG, Moya FR, et al.: A pilot, randomized, controlled clinical trial of lucinactant, a peptide-containing synthetic surfactant, in infants with acute hypoxemic respiratory failure. *Pediatric Critical Care Medicine* 2012; 13:646–653

222. Tibballs J, Shann FA, Landau LI: Placebo-controlled trial of prednisolone in children intubated for croup. *The Lancet* 1992; 340:745–748

223. Tibby SM, Hatherill M, Wright SM, et al.: Exogenous surfactant supplementation in infants with respiratory syncytial virus bronchiolitis. *American Journal of Respiratory and Critical Care Medicine* 2000; 162:1251–1256

224. Torres S, Sticco N, Bosch JJ, et al.: Effectiveness of magnesium sulfate as initial treatment of acute severe asthma in children, conducted in a tertiary-level university hospital. a randomized, controlled trial. *Arch Argent Pediatr* 2012; 110:291–296

225. Umenai T, Shime N, Hashimoto S: Hyperventilation versus standard ventilation for infants in postoperative care for congenital heart defects with pulmonary hypertension. *J Anesth* 2009; 23:80–86

226. Upadhyay M, Singhi S, Murlidharan J, et al.: Randomized evaluation of fluid resuscitation with crystalloid (saline) and colloid (polymer from degraded gelatin in saline) in pediatric septic shock. *Indian Pediatr* 2005; 42:223–231

227. Upadhyay P, Tripathi VN, Singh RP, et al.: Role of hypertonic saline and mannitol in the management of raised intracranial pressure in children: a randomized comparative study. *J Pediatr Neurosci* 2010; 5:18

## Appendix B: Included Trials


228. Valoor HT, Singhi S, Jayashree M: Low-dose hydrocortisone in pediatric septic shock: An exploratory study in a third world setting. *Pediatric Critical Care Medicine* 2009; 10:121–125

229. Vardi A, Salem Y, Padeh S, et al.: Is propofol safe for procedural sedation in children? A prospective evaluation of propofol versus ketamine in pediatric critical care. *Critical Care Medicine* 2002; 30:1231–1236

230. Venter M, Rode H, Sive A, et al.: Enteral resuscitation and early enteral feeding in children with major burns—effect on McFarlane response to stress. *Burns* 2007; 33:464–471

231. Vlasselaers D, Milants I, Desmet L, et al.: Intensive insulin therapy for patients in paediatric intensive care: a prospective, randomised controlled study. *The Lancet* 2009; 373:547–556

232. van Waardenburg DA, de Betue CT, van Goudoever JB, et al.: Critically ill infants benefit from early administration of protein and energy-enriched formula: a randomized controlled trial. *Clinical Nutrition* 2009; 28:249–255

233. Wheeler DS, Jacobs BR, Kenreigh CA, et al.: Theophylline versus terbutaline in treating critically ill children with status asthmaticus: a prospective, randomized, controlled trial. *Pediatric Critical Care Medicine* 2005; 6:142–147

234. Willson DF, Zaritsky A, Bauman LA, et al.: Instillation of calf lung surfactant extract (calfactant) is beneficial in pediatric acute hypoxemic respiratory failure. *Critical Care Medicine* 1999; 27:188–195

235. Willson DF, Thomas NJ, Markovitz BP, et al.: Effect of exogenous surfactant (calfactant) in pediatric acute lung injury: a randomized controlled trial. *JAMA* 2005; 293:470–476

236. van Woensel JBM, van Aalderen WMC, de Weerd W, et al.: Dexamethasone for treatment of patients mechanically ventilated for lower respiratory tract infection caused by respiratory syncytial virus. *Thorax* 2003; 58:383–387

237. van Woensel JBM, Vyas H, STAR Trial Group: Dexamethasone in children mechanically ventilated for lower respiratory tract infection caused by respiratory syncytial virus: a randomized controlled trial. *Critical Care Medicine* 2011; 39:1779–1783

238. Yañez LJ, Yunge M, Emilfork M, et al.: A prospective, randomized, controlled trial of noninvasive ventilation in pediatric acute respiratory failure. *Pediatric Critical Care Medicine* 2008; 9:484–489

239. Yildizdas D, Yapicioglu H, Yilmaz HL: Occurrence of ventilator-associated pneumonia in mechanically ventilated pediatric intensive care patients during stress ulcer prophylaxis with sucralfate, ranitidine, and omeprazole. *J Crit Care* 2002; 17:240–245

240. Yildizdas D, Yapicioglu H, Celik U, et al.: Terlipressin as a rescue therapy for catecholamine-resistant septic shock in children. *Intensive Care Med* 2007; 34:511–517

241. Yildizdas D, Yapicioglu H, Tümgör G, et al.: Çocuk yoğun bakım ünitesi'nde sepsis nedeni ile izlenen hastalarda poliklonal intravenöz immünglobülin tedavisi mortaliteyi azaltıyor mu? *Cocuk Sagligi Ve Hastaliklari Dergisi* 2005; 48:136–141

242. Ying-qian Z, Bo H, Jian-hua L, et al.: Efficacy of ciprofloxacin for the treatment of pediatric severe infections. *Chinese Journal of Antibiotics* 2005; 30:416

243. Yu K-Y, Huang X-H, Li H-D: [Clinical observation on treatment of gastrointestinal dysfunction by fu'an liquid for retention enema in children with critical illness]. *Zhongguo Zhong Xi Yi Jie He Za Zhi* 2002; 22:261–263

244. Yung M, Keeley S: Randomised controlled trial of intravenous maintenance fluids. *Journal of Paediatrics and Child Health* 2009; 45:9–14

245. Zhao K, Wang W, Zhang J, et al.: Effects of high-dose mucosolvin on lung functions in infant patients with cardiopulmonary bypass. *Heart Surg Forum* 2011; 14:E227–31

246. Zhao S-K: [Nasal continuous positive airway pressure for the treatment of severe pneumonia in children from the plateau area: clinical analysis of 47 cases]. *Zhongguo Dang Dai Er Ke Za Zhi* 2010; 12:226–227

247. Zobel G, Kuttnig M, Grubbauer HM, et al.: Reduction of colonization and infection rate during pediatric intensive care by selective decontamination of the digestive tract. *Critical Care Medicine* 1991; 19:1242–1246
